# The kynurenine pathway in HIV, frailty and inflammaging

**DOI:** 10.3389/fimmu.2023.1244622

**Published:** 2023-09-08

**Authors:** Shabiha Sultana, Anthony Elengickal, Husam Bensreti, Eric Belin de Chantemèle, Meghan E. McGee-Lawrence, Mark W. Hamrick

**Affiliations:** Medical College of Georgia, Augusta University, Augusta, GA, United States

**Keywords:** inflammation, aryl hydrocarbon receptor, IDO1, sarcopenia, osteoporosis

## Abstract

Kynurenine (Kyn) is a circulating tryptophan (Trp) catabolite generated by enzymes including IDO1 that are induced by inflammatory cytokines such as interferon-gamma. Kyn levels in circulation increase with age and Kyn is implicated in several age-related disorders including neurodegeneration, osteoporosis, and sarcopenia. Importantly, Kyn increases with progressive disease in HIV patients, and antiretroviral therapy does not normalize IDO1 activity in these subjects. Kyn is now recognized as an endogenous agonist of the aryl hydrocarbon receptor, and AhR activation itself has been found to induce muscle atrophy, increase the activity of bone-resorbing osteoclasts, decrease matrix formation by osteoblasts, and lead to senescence of bone marrow stem cells. Several IDO1 and AhR inhibitors are now in clinical trials as potential cancer therapies. We propose that some of these drugs may be repurposed to improve musculoskeletal health in older adults living with HIV.

## 1 Kynurenine accumulation with aging and HIV infection

The World Health Organization estimates that 38 million people are infected with HIV worldwide, and of those 23 million currently receive antiretroviral therapy (ART). Progress in ART has enabled patients to live longer, and approximately 50% of people with HIV are older than 50 years (1). Yet, patients on ART still experience major complications and comorbidities from HIV infection. It is estimated that 20-30% of patients receiving ART experience significant muscle wasting (2, 3). Indeed, a frailty phenotype is frequently observed in HIV patients on long-term ART (4), involving declines in functional measures such as grip strength and gait speed (5, 6). In addition, both osteoporosis and sarcopenia are often observed together in older people living with HIV (7). Loss of muscle mass and strength are in turn associated with poor health outcomes ranging from accelerated disease progression to increased mortality (8, 9). The mechanisms underlying impaired muscle function and the frailty phenotype of patients with HIV are not well understood, but they do suggest an earlier onset of age-related musculoskeletal decline as compared to the HIV-naïve population, a phenomenon referred to as accentuated aging (10). A key question from the recent (2020) NIH HIV ACTION Workshop on Pathogenesis of Aging in People with HIV (10) is “Do the mechanisms and mediators of age-related end-organ disease (e.g., sarcopenia) differ in aging with and without HIV and ART?”

Tryptophan (Trp) is an essential amino acid that cannot be synthesized in humans and is only available through dietary sources. Approximately 90-95% of dietary Trp is degraded along the kynurenine (Kyn) pathway by two major enzymes; tryptophan 2, 3 dioxygenase (TDO) in the liver, and indoleamine 2, 3 dioxygenase (IDO) extrahepatically (11). A smaller (~3%) portion of Trp is metabolized along the serotonin/melatonin pathway. In the kynurenine pathway, Kyn is the first stable intermediate metabolite formed (12). Kyn has been reported to exert its functions through the Aryl hydrocarbon receptor (AhR), which acts as a transcription factor (13, 14). A number of recent studies demonstrate that Kyn increases with age (15–17). For example, Sorgdrager et al. (15) demonstrated that Kyn is increased in serum with age in humans but Kyn metabolites such as kynurenic acid (KA) and quinolinic acid (QA) also increase, suggesting that the entire Kyn pathway may be upregulated with aging. The authors found that the same metabolites also increased with age in the cerebrospinal fluid (15). Likewise, de Bie et al. showed that the Kyn/Trp ratio was significantly correlated with age in older women (16), and El Refaey et al. showed that in mice Kyn increased in an age-dependent manner (17).

Kyn has been implicated in several age-related disorders including neurodegeneration, osteoporosis, sarcopenia, and inflammation (16–20). Treatment of rodents with exogenous Kyn, either through diet or intraperitoneal injections, can induce bone loss (17, 21, 22) and muscle atrophy (19). An increase in IDO activity has been linked to an increased mortality rate in humans (23), and frailty is associated with a marked increase in the Kyn/Trp ratio (24–27). Suppressing Trp degradation and thus Kyn accumulation increases lifespan in both *C. elegans* (28) and *Drosophila* (29), and increased longevity in bats is associated with endogenous inhibition of Trp breakdown by the gut microbiome (30). Importantly, Kyn increases with progressive disease in HIV patients (31–34) along with increased serum IFN gamma (35). Furthermore, ART does not normalize IDO activity in these subjects (36, 37).

## 2 Mechanisms of kynurenine biogenesis with aging and HIV infection

**Figure 1:**
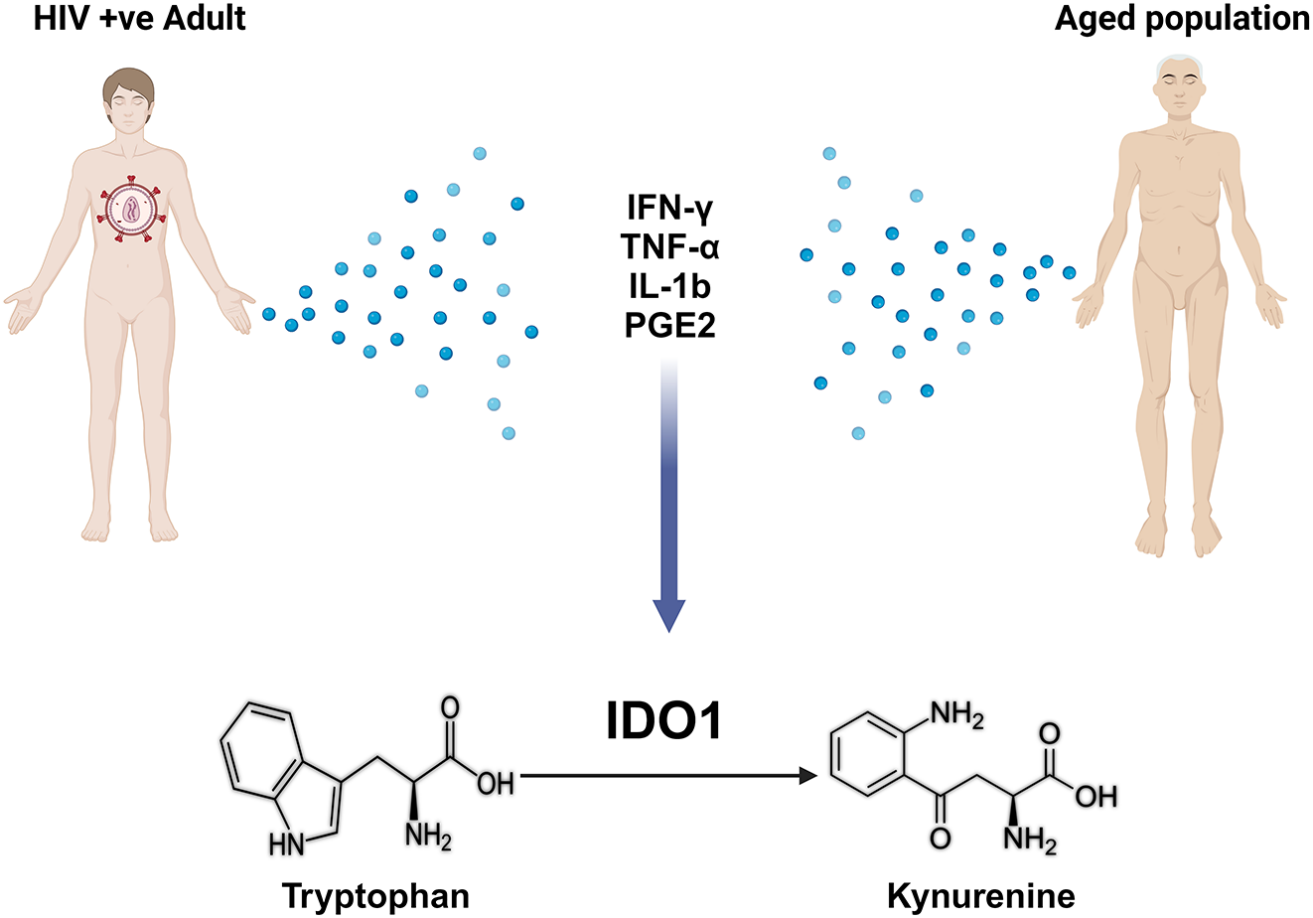
Shared mechanisms between aging and HIV infection in enhancing tryptophan metabolism to kynurenine via IDO1. Figure generated using BioRender.

Trp commitment to the Kyn pathway is mediated by indoleamine 2,3-dioxygenase (IDO1/IDO2) or tryptophan 2,3-dioxygenase 2 (TDO) (38–40). The expression of TDO and IDO enzymes in mammals varies not only by tissue/cellular localization but also by the mechanism of their stimulation (12). TDO is primarily found in the liver and neuronal tissue and is regulated by glucocorticoids, where it helps to maintain homeostasis of dietary Trp levels and mediate immune-related diseases and central nervous system disorders (41) The function of IDO1 was initially described as an innate mechanism of defense against microbial invasion because IDO1 could induce depletion of Trp (41). The expression and activity of IDO1 are upregulated in response to several inflammatory mediators including interferon-gamma (IFN-γ) (42), interleukin-1-beta (IL-1β) (43), tumor necrosis factor (TNF- α) (42), and prostaglandin-E2 (PGE2) (44). A substantial increase in inflammatory mediators such as IFN-γ, IL-1β, and TNF- α is seen in various organs and tissues with aging, a process termed inflammaging (45–48). The increase in these inflammatory cytokines increases TDO and IDO expression, further catabolizing Trp and increasing the accumulation of Kyn pathway metabolites (38). HIV infection has also been shown to increase inflammatory mediators enhancing IDO1 expression (49–51). Furthermore, it has been observed that HIV-infected individuals have lower circulating Trp levels despite adequate Trp dietary intake, suggesting an enhanced Trp degradation (52). This common mechanism in IDO1 activation in both aging and HIV infection might shed some light on the observed accelerated aging in HIV infected individuals (53) (**Figure 1**). Nuclear factor kappa B (NF-κB) activation downstream of inflammatory cytokine signaling is known to induce TDOI/DO1 (31–33, 54), which is considered among the most common mechanisms of Kyn biogenesis in the setting of systemic inflammation with aging and HIV infection.

The association between the kynurenine pathway and human immunodeficiency virus (HIV) infection has been established since 1991, when the elevated kynurenine-to-tryptophan (Kyn/Trp) ratio was observed in people living with HIV (PWH) (30). Moreover, it was also observed that, after the initial stage of infection, people receiving early ART demonstrated a rapid return of the Kyn/Trp ratio to normal concomitant with an improvement in CD4+/CD8+T cells ratio in accordance with lower mucosal inflammation (55). This observation suggested a correlation between heightened kynurenine pathway activity and compromised immune function in the context of HIV infection (33). Dendritic cells (DCs) and monocytes express the enzyme IDO-1, which catabolizes Trp into Kyn. This alters CD4+ T-cell development into regulatory T cells (Tregs) rather than T-helper (Th17) cells, which negatively affects T-cell responses. Through interferon signaling and Toll-like receptor stimulation, this altered Th17/Treg balance is directly related to elevated and sustained IDO-1 activity (56). Increased IDO-1 activity is further associated with the plasma microbial translocation markers, and the progression of HIV (57). Immunological dysfunction and ongoing immunological activation during HIV infection are both known to be significantly influenced by microbial translocation (58). A link may also exist between IDO activity and total HIV DNA in blood, pointing to a potential role for IDO in HIV persistence (57). There is also evidence that, in the case of HIV infection, gut dysbiosis is a major driver of IDO1 activity, Trp degradation, and Kyn biogenesis (59, 60). Specifically, the HIV infection itself can cause pronounced changes to the gut microbiota, leading to increased Trp degradation and elevated Kyn accumulation independent of IFN-gamma or NF-kB activity (35, 61, 62). The activation of the kynurenine pathway has been observed to occur through the generation of neurotoxic quinolinic acid in macrophages, as a direct result of the actions of the HIV proteins Tat, Nef, and gp41 (63).

## 3 Role of kynurenine signaling through the aryl hydrocarbon receptor in muscle and bone loss with aging and HIV infection

**Figure 2:**
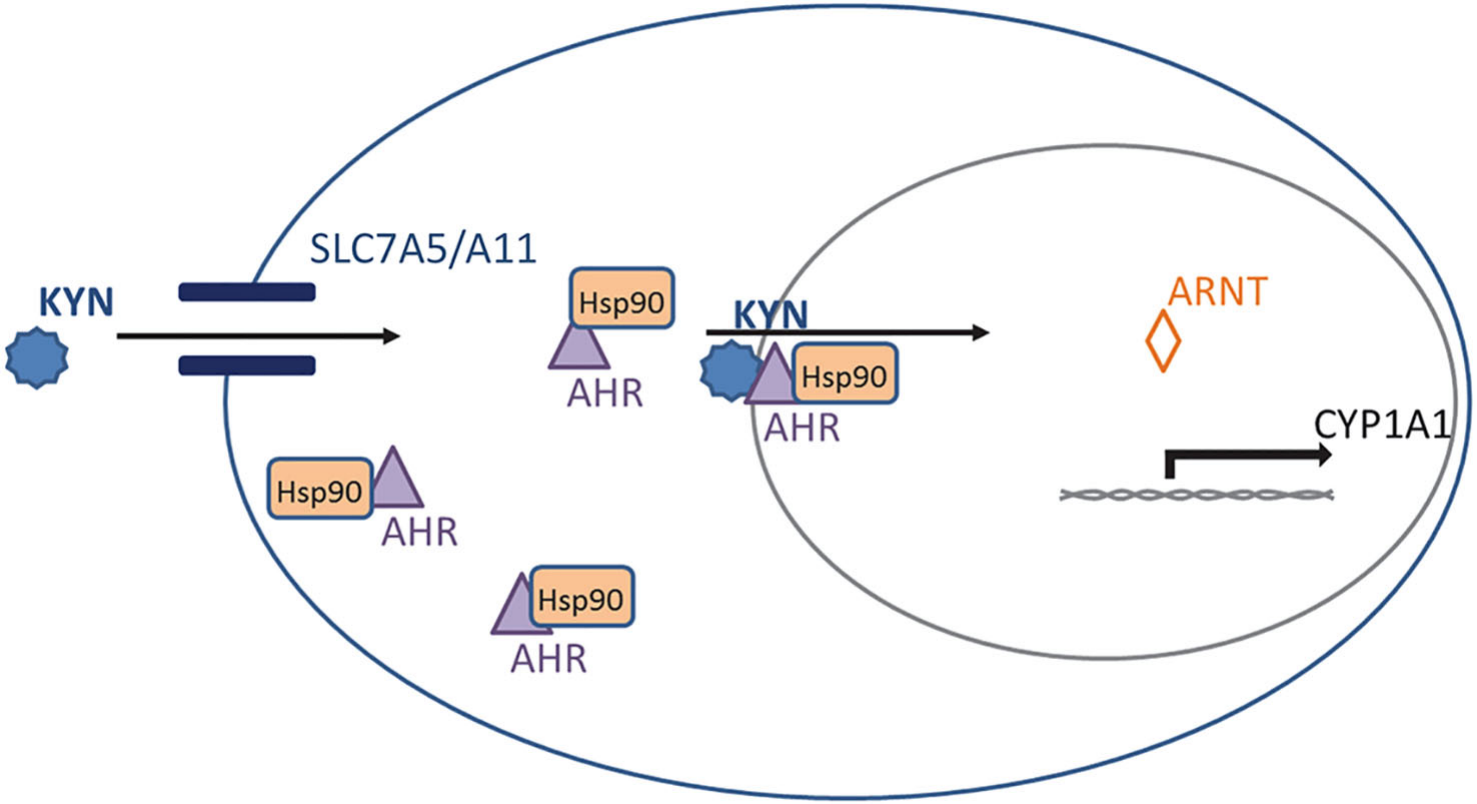
Kynurenine (Kyn) signaling through the aryl hydrocarbon receptor (AhR). AhR is normally bound to heat-shock protein 90 (Hsp90) in the cytoplasm. Kyn enters the cell via transporters SLC7A5 and SLC7A11 and binds the Hsp90-AhR complex. This complex translocates to the nucleus where AhR forms a complex with aryl hydrocarbon receptor nuclear translocator (Arnt) protein to activate downstream targets such as Cyp1a1.

Kyn is an endogenous ligand of the aryl hydrocarbon receptor (AhR) (14, 23, 24, 64). AhR is bound to the molecular chaperone heat-shock protein 90 (Hsp90) and dwells there in an inactive state in the cytoplasm of the cell. Trp and its metabolite Kyn both enter the cell via transporters including solute carrier family 7 member 5 (SLC7A5) and member 11 (SLC7A11) (65) (**Figure 2**). Once inside the cell, Kyn can stimulate AhR translocation to the nucleus (66, 67)where AhR separates from Hsp90 and forms a dimer with the aryl hydrocarbon receptor nuclear translocator (ARNT) protein. The heterodimer binds to a specific DNA sequence called the xenobiotic response element, or XRE, in the promoter region of potential target genes (**Figure 2**). Previously it has been shown that Kyn’s AhR and transcriptional activation of AhR stimulates both TGF beta 1 and IDO1 expression; this creates a positive feedback loop as IDO1 can degrade tryptophan to produce more Kyn (68, 69). Moreover, AhR has been demonstrated to upregulate the expression of SLC7A5 (70, 71). Thus, the increase in SLC7A5 with kynurenine-activated Ahr probably facilitates more kynurenine entry into the cell (72).

In cases of viral infection, constitutive AhR signaling inhibits the type I interferon (IFN-I) response by upregulating the ADP-ribosylase TIPARP (73). A recent study found that AhR activation by Trp metabolites, especially Kyn, promoted HIV infection and decreased CD4+ T cell counts. AhR activates viral transcription and infection by directly binding to the HIV-1 5′ Long terminal repeat (5′-LTR) (74). Moreover, Trp metabolites increase AhR translocation to the nucleus which leads to the association with HIV 5′-LTR. Further, AhR and the HIV-1 Tat protein may bind, attracting favorable transcriptional elements that aid in infection (74). However, the role of AhR on HIV-1 activation may vary according to cell type. For example, another study found that, in macrophages, the activation of AhR is responsible for blocking HIV-1 replication by downregulating the transcription of cyclin-dependent kinase CDK1, CDK2 which ultimately exerts antiviral effects (75). Moreover, long-term exposure to AhR ligands induces hypomethylation of AhR DNA binding regions (76), and HIV infection increased hypomethylation in the promoters of numerous genes including AhR (77).

**Figure 3:**
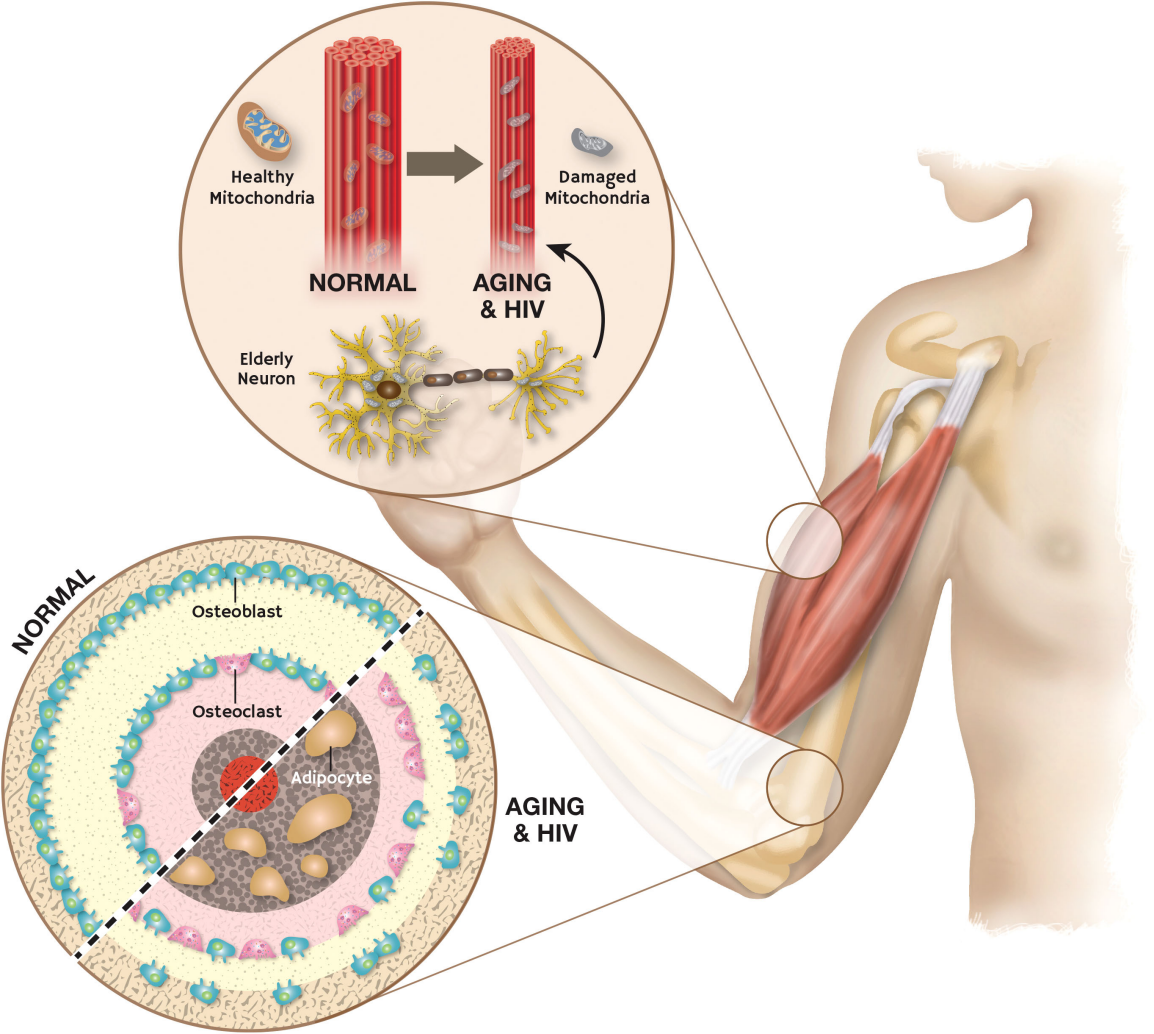
Aging and HIV induced damage in skeletal muscle and dysregulation of bone homeostasis: Aging and HIV cause mitochondrial damage in skeletal muscle which leads to frailty. Activation of AhR in aging and HIV causes disruption of bone homeostasis and fat deposition in skeletal muscle.

Muscle loss is frequently observed in HIV patients on ART which contributes directly to increased morbidity (2). The clinical signs of frailty, such as loss of energy, weight, and muscle mass, as well as reduced motor function and low levels of physical activity, are strikingly similar to those of advanced HIV infection (78). A key frailty mediator is a mitochondrial dysfunction which increases with aging (79). Hunt et al. (80) showed that PWH receiving contemporary ART have more skeletal muscle mitochondrial impairment than would be expected based on age alone. Activation of AhR by Kyn in the setting of aging and HIV infection is very likely a drive of muscle dysfunction in these patients. We have found that Kyn treatment in younger mice can reduce muscle size and strength with an increase in reactive oxygen species levels (19). On other hand, inhibition of IDO can increase muscle size and strength in aged mice (19). These findings are further supported by work showing that constitutive AhR overexpression increases ROS accumulation, mitochondrial dysfunction, and skeletal muscle atrophy (81). Aging and HIV infection therefore share several biological characteristics associated with musculoskeletal frailty (**Figure 3**).

Similar findings have been made in bone that point to Kyn-induced AhR activation as a driver of bone dysfunction with aging and HIV infection. Bone mineral density (BMD) can decline rapidly in patients with HIV starting ART (82). Moreover, BMD in HIV-positive women decreases twice as quickly as compared to HIV-positive men (82). Activation of AhR signaling promotes osteoporosis with aging by stimulating the formation of osteoclasts (83, 84). While there is abundant research supporting the involvement of Kyn involvement in bone loss (17, 85–87), Eisa NH., et al. (13) further clarified that osteoclastic transcription factors are upregulated by Kyn signaling through AhR which results in an increased number of osteoclasts. Moreover, Kyn treatment in mice caused an increased serum level of the osteoclastic marker RANKL and the bone resorption marker Pyd (17). Another study reported that activation of AhR impeded the proliferation of osteoblasts derived from human mesenchymal stem cells (85), and we recently showed that AhR activation induced senescence in bone marrow stem cells (86). Kim and colleagues (87) showed the detrimental effects of Kyn on human bone metabolism where they studied bone marrow (BM) samples from patients undergoing hip surgery. They found that BM kynurenine levels increased with age and it was linked to higher fragility hip fracture risk, an increase in TRAP-5b and RANKL (two markers of bone resorption) and a decline in total femur bone mineral density (BMD). Together, these findings suggest that AhR activation in the setting of HIV infection promotes both muscle and bone loss with aging.

## 4 Potential strategies for therapeutic intervention

A number of experimental therapies that target the Kyn pathway are currently being tested as novel treatments for various cancers (**Table 1**). The majority of these, such as Epacadostat, Indoximod and Linrodostat are inhibitors of IDO1 that blunt the formation of Kyn. Others such as IK-175 and BAY2416964 are direct inhibitors of AhR activity. These therapeutics have been reported to improve outcomes in cancer patients across several different clinical trials and have shown better efficacy when used in combination with other therapeutics such as Pembrolizumab, an immune checkpoint inhibitor (CTI) (88).

**Table 1:** Inhibitors in clinical trials targeting the Kyn pathway in cancer therapeutics.

Drug	Mechanism of Action	Status	NCT Numbers
Epacadostat	IDO1 Inhibitor	Phase 3 Trials	NCT03361865 NCT03374488 NCT03260894 NCT03358472 NCT02752074
Linrodostat	IDO1 Inhibitor	Phase 3 trials	NCT03329846 NCT03661320
Indoximod	IDO Inhibitor	Phase 2 Trials	NCT01560923 NCT01792050 NCT04049669 NCT02077881
IK-175	AhR Inhibitor	Phase 1 Trials	NCT04200963 NCT05472506
BAY2416964	AhR Inhibitor	Phase 1 Trials	NCT04999202 NCT04069026
HTI-1090	IDO1 and TDO Inhibitor	Phase 1 Trials	NCT03491631 NCT03208959

Epacadostat, a highly selective and orally available inhibitor of IDO1, is currently being studied in phase 3 clinical trials following positive results from multiple phase 1 and 2 studies for a variety of cancers. In one phase 1/2 study, Epacadostat was used in combination with Pembrolizumab for patients with advanced solid tumors and showed antitumor activity and safety (89). Similar results were obtained in another phase 1/2 study of Epacadostat used with ipilimumab in patients with melanoma (90). Linrodostat is another oral inhibitor of IDO1 currently in phase 3 trials with a slightly different mechanism of action, functioning as an irreversible suicide inhibitor. In a phase 1/2 study of patients with advanced bladder cancer, the combination of nivolumab and Linrodostat was well tolerated and showed efficacy across the patient population (91). Indoximod is a Trp analogue that can suppress the IDO pathway which is normally upregulated in the absence of Trp. After demonstrating safety and efficacy in phase 1 trials of patients with advanced solid tumors (92, 93), many phase 2 clinical trials are currently active. One of these trials that have published results was a phase 2 trial investigating the addition of indoximod to patients with advanced melanoma receiving CTIs such as pembrolizumab. The therapeutic was well tolerated in this patient population and showed antitumor efficacy (94). **Table 1** summarizes some of the inhibitors in clinical trials targeting AhR/IDO in cancer therapeutics.

## 5 Summary & conclusions

HIV is associated with accelerated or accentuated frailty. This is characterized by aging phenotypes such as cognitive impairment, cardiovascular disease, and muscle and bone loss. HIV-related proteins themselves, as well as cytokines such as IFN-gamma, increase expression of the Trp degrading enzyme IDO1, generating Kyn as a by-product of Trp degradation. Kyn and the Kyn/Trp ratio are markedly elevated in patients with HIV, even after antiretroviral therapy. Similarly, aging causes an increase in inflammatory mediators that upregulate activity of IDO1, thus promoting Trp metabolism to Kyn. However, IDO1 activity may be more pronounced in HIV infection due to gut dysbiosis which may drive IDO1 activity. Increased IDO1 is associated with microbial translocation and progression of HIV. Kyn induces senescence in bone marrow cells, reduces bone formation, and contributes to muscle atrophy and damage to the neuromuscular junction. These effects appear to be mediated in part by activation of aryl hydrocarbon receptor signaling. Small molecules that suppress Trp degradation by IDO1 have shown potential for synergizing with antiretroviral therapies to improve health outcomes. Future studies should be directed at examining AhR inhibition as a potential companion strategy.

## Author contributions

SS, AE, and HB prepared the initial draft, Table and **Figure 1**. MH, EB and MM-L prepared **Figure 2** and the subsequent draft revisions. All authors contributed to the article and approved the submitted version.
